# Diet change and exercise enhance protein expression of CREB, CRTC 2 and lipolitic enzymes in adipocytes of obese mice

**DOI:** 10.1186/s12944-016-0316-2

**Published:** 2016-09-05

**Authors:** Jinhee Woo, Sunghwun Kang

**Affiliations:** 1 Laboratory of Exercise Physiology, Department of Physical Education, Dong-A University, Hadan-dong, Saha-gu, 49315, Busan, 604-714 Republic of Korea; 2 Laboratory of Exercise Physiology, Division of Sport Science, Kangwon National University, 1 Kangwondaehak-gil, Chuncheon-si, Gangwon-do 24341 Republic of Korea

**Keywords:** CREB, CRTC, High fat diet, Exercise, Diet change

## Abstract

**Background:**

The study investigated the effects of regular exercise and diet changes on the change in metabolic processes of the cAMP-Response Element-Binding Protein-Regulated Transcription Coactivator (CRTC) family and its sub-lipolysis.

**Methods:**

Four-week-old C57/black male mice received an 8-week diet of general formula (control, CO; *n* = 10) or a high fat diet (HF; *n* = 30) to induce obesity. Thereafter, the mice received another 8-week regimen of general formula CO (*n* = 10) diet, continuous HF diet (*n* = 10), switched to general formula (diet change, DC; *n* = 10) or switched to general formula + exercise (diet and exercise, DE; *n* = 10).

**Results:**

The DE group displayed significantly lower body weight, abdominal fat and lipid profiles (*p* < 0.05). The DE group also displayed significantly lower (35 %) CRTC 2 activity than the CO (*p* < 0.05). Activities of adipose triglyceride lipase (ATGL), hormone sensitive lipolitic enzyme (HSL) and monoacylglycerol lipase (MGL) were significantly higher (51 %, 38 %, 49 %) in the DE group than the HF group (*p* < 0.05). MGL, there were no differences between the CO group, HF group, and DC group, with the DE group (70 %) being significantly higher (*p* < 0.05).

**Conclusion:**

Change in diet in the absence of exercise was not associated with changes in adipose tissue CRTC family lipase activity, indicating that lipolysis metabolic processes are effective only when diet and exercise are carried out together.

## Background

The cAMP-response element-binding protein (CREB)-regulated transcription coactivator (CRTC) family comprises three functionally diverse proteins: CRTC 1, 2 and 3. CRTC 1 may be related to reducing energy consumption by virtue of control of the expression of leptin in the hypothalamus [1]. CRTC 2 facilitates insulin resistance by triggering gluconeogenesis in the liver in conjunction with CREB during fasting in obese individuals [2, 3]. CRTC 3, which has been termed an energy saving gene, participates in the inhibition of fat catabolism and lipolysis, which is important in survival during periods of food scarcity, by association with catecholamine in energy balance [4].

Activation of the gene encoding CRTC 3 inhibits lipolysis that is likely to result in obesity. However, energy consumption was increased despite the induction of obesity in CRTC 3 knockout mice consuming a high calorie diet. In addition CRTC 3 knockout mice increases in the quantity and functioning of brown fat compared to the control group [4].

Triacylglycerol (TAG) is hydrolyzed through the activities of a trio of enzymes: adipose triglyceride lipase (ATGL), hormone sensitive lipase (HSL) and monoacylglycerol lipase (MGL) [5]. Catecholamine is essential in TAG hydrolysis as a supra-regulator. Catecholamine couples to β-adrenergic receptors in white adipocytes and subsequently stimulates adenylyl cyclase catalyzing the production of cyclic AMP (cAMP). The increased cAMP stimulates protein kinase A (PKA), ultimately activate HSL. While lipolysis TAG is hydrolyzed by ATGL catalysis and converted to diacylglycerol (DAG), which is in turn converted to monoacylglycerol (MAG) by HSL. MAG enters the circulatory system where it is degraded to the fatty acid and glycerol [6].

Regular exercise reduces fat volume through energy consumption [7]. Secretion of catecholamines induces the series of enzyme-mediated activities described above that lead to the fatty acid and glycerol degradation products. Even though catalyzed sub-lipolysis metabolic process is closely related with energy balance, there is no investigation about the exercise related metabolic mechanism as well as the difference between body fat reduction mediated by diet change and exercise. This study was undertaken to determine the effects of diet change and exercise on the CRTC family and the activity level of the lipolysis enzyme in the white fat of obese mice.

## Methods

### Animals

Four-week-old C57/Black male mice were assigned to an 8-week regimen of a control diet (fat 6.25 %, protein 24.34 %, carbohydrate 69.41 %) group (CO; *n* = 10) or high fat diet group (HF; *n* = 30). The HF diet (fat 60 %, protein 20 % and carbohydrate 20 %, Research diet, Inc D12492) induced obesity for 8 weeks. Thereafter, they were assigned to an 8-week CO group (*n* = 10), HF group (*n* = 10), diet change (fat 6.25 %, protein 24.34 %, carbohydrate 69.41 %) group (DC, *n* = 10), and a diet change + exercise group (DE, *n* = 10). The laboratory animals were caged (3 or 4 per cage) for breeding at a temperature of 22–24 °C, 60 % humidity and alternating 12-h light and dark periods. CO mice were provided with solid feed (Samtako Bio Korea, Korea) corresponding to AIN-76 (American Institute of Nutrition, 1977) and were allowed water as desired. The obesity groups (HF, DC and DE) were also provided the HF diet and water for 8 weeks after a 1-week environmental adaptation. The study was carried out in accordance with the Guidelines laid down by the NIH in the US regarding the care and use of animals for experimental procedures and was approved by DONG-A University Institutional Care and Use Committee (Dong-A Univ. Hospital, Korea).

### Exercise program

The exercise component of the DE group consisted of 30 min per day, 5 days per week sessions on a mouse treadmill for 8 weeks. Treadmill exercise was conducted under these fixed conditions with mild-intensity at 0 ° inclination from weeks 1 to 4, with the exercise intensity set at 5 m/min for the first 5 min, then at 10 m/min for the next 30 min and at 5 m/min for the last 5 min. From weeks 5 to 8, moderate intensity exercise involved a treadmill speed of 5 m/min for the first 5 min, 14 m/min for the next 30 min and 5 m/min for the last 5 min [8].

### Blood and tissue analysis

Blood and tissue samples were acquired 48 h after the end of exercise to rule out the transient effects of the exercise in the case of the DE group. During the sampling, the feed supply was stopped 12 h before sampling but water supply continued. All laboratory animals were anesthetized with ethyl ether and the blood samples were collected from the abdominal inferior vena cava, and then the abdominal visceral fat was extracted and weighed. The extracted fat was stored at -80 °C until analysis. Plasma total cholesterol (TC), triglyceride (TG) levels and high density lipoprotein cholesterol (HDL-c) concentrations were measured (TC, TG, HDL-c kits, Asan Pharmaceutical, Korea) on Sunrise automatic biochemistry analyzer (TEKAN, Switzerland) by the enzymatic colorimetric method using commercially available radioimmunoassay kit (Mercodia, Sweden). The low density lipoprotein cholesterol (LDL-c) was calculated by the formula described by Friedwald, Levy & Fredrickson [9]:

$$ \mathrm{L}\mathrm{D}\mathrm{L}\hbox{-} \mathrm{c} = \mathrm{T}\mathrm{C}\ \hbox{--}\ \left(\mathrm{H}\mathrm{D}\mathrm{L}\hbox{-} \mathrm{c} + \mathrm{T}\mathrm{G}/5\right) $$

The serum glucose level was estimated using a GlucoDr glucometer (Allmedicus, Korea). Plasma insulin level was determined spectrophotometrically with a rat insulin ELISA kit (Mercodia, Sweden) according to the manufacturer’s instructions. Insulin resistance index was assessed by homeostasis model assessment estimate of insulin resistance (HOMA-IR) as follows:

$$ \mathrm{HOMA}\hbox{-} \mathrm{I}\mathrm{R} = \mathrm{Fasting}\ \mathrm{insulin}\ \left(\upmu \mathrm{I}\mathrm{U}/\mathrm{mL}\right) \times \mathrm{Fasting}\ \mathrm{glucose}\ \left(\mathrm{mg}/\mathrm{dL}\right)/405 $$

To isolate protein from the adipose tissue, 200 μl of radioimmunoprecipitation assay (RIPA) buffer [50 mM Tris-HCl, pH 8.0, with 150 mM sodium chloride, 1.0 % Igepal CA-630 (NP-40), 0.5 % sodium deoxycholate, 0.1 % sodium dodecyl sulfate, protease inhibitor cocktail and phosphatase inhibitor cocktail] was added. The tissue was homogenized and adipose tissue was lysed by incubation on ice for 30 min. The lysed adipose tissue was centrifuged at 13,000 rpm at 4 °C for 30 min, and the supernatant was transferred to a clean e-tube and stored at -80 °C. Western blotting used protein quantified using the BCA protein assay kit (PIERCE, USA). The protein was subjected to 12 ~ 15 % sodium dodecyl sulfate-polyacrylamide gel electrophoresis (SDS-PAGE) at 100 V. The membrane was blocked using 5 % skim milk prior to reaction overnight at 4 °C with primary antibody to CRTC 2 (SC-271912), CRTC 3 (SC-139569), ATGL (SC-50223), or HSL (SC-25843) (all from Santa Cruz Biotechnology, USA) or MGL (ab24701) (Abcam, UK), followed by the secondary antibody reaction for 1 h at room temperature. Bound antibody was visualized by ECL solution (Amersham Pharmacia Biotech, USA) and the expression of protein was confirmed using ImageQuant™ LAS-4000 (GE Healthcare, SWE)

### Data analysis

Mean and standard error were calculated for all measured items using SPSS Windows Ver. 20.0 statistical package (SPSS, USA). Two-way ANOVA was used to verify the inter-times and the inter-group weight differences. One-way ANOVA was used to verify the inter-group differences in the blood components and the results of tissue analysis. When statistical significance was evident, Duncan’s post hoc analysis was carried out. Statistical significance was set as ⍺ = 0.05

## Results

**Fig. 1:**
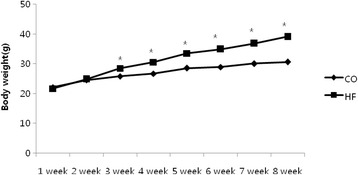
Change of body weight during 8-week high fat diet. Mean ± SE, *p < 0.05 vs CO

**Fig. 2:**
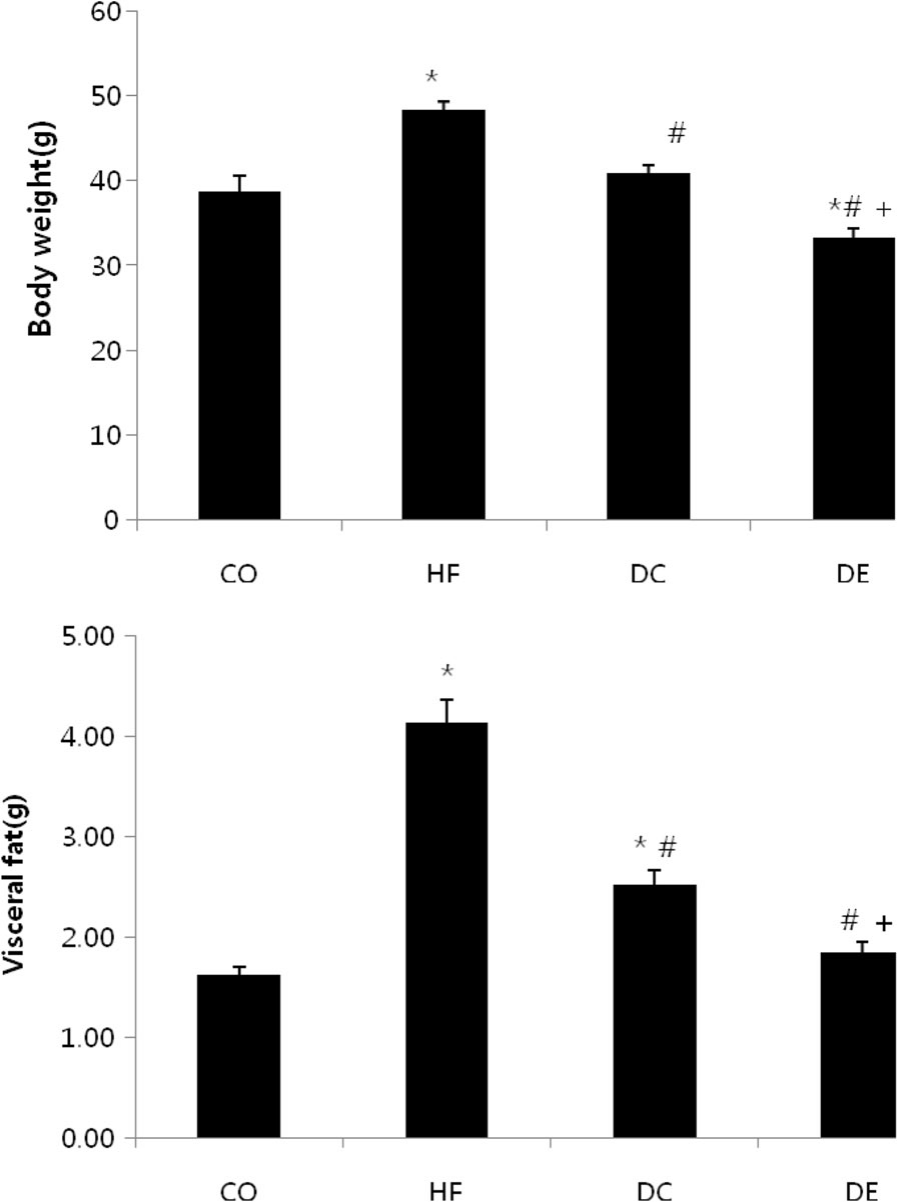
Different of body weight and visceral fat after exercise and diet. Mean ± SE, *p < 0.05 vs CO, #*p* < 0.05 vs HF, +*p* < 0.05 vs DC

There was no difference in body weight between the CO and HF group before start high fat diet. The body weight in the HF group significantly increased after 3 weeks high fat diet (Fig. 1). Body weight and visceral fat after 8 weeks was significnat decreased in the DC and DE group compared with the HF group (*p* < 0.05) (Fig. 2).

Body weight and abdominal fat volume were significantly greater in the HF group than the other groups (*p* < 0.05), and in the DE group compared to the DC group (*p* < 0.05). Among the blood components, total cholesterol (TC), high-density lipoprotein cholesterol, low-density lipoprotein cholesterol (LDLC), insulin and homeostatic model assessment-insulin resistance (HOMA-IR) were significantly different in the DE group compared to the HF group (*p* < 0.05). TC, LDLC, insulin, and HOMA-IR were significantly different in the DC group compared to the HF group (*p* < 0.05; Table 1).

**Table 1:** Change of insulin resistance and lipid profiles after exercise and diet

Variable	CO	HF	DC	DE
Glucose (mg/dL)	125.60 ± 9.79	182.40 ± 7.64*	150.00 ± 12.96	148.00 ± 7.15
TG (mmol/L)	0.46 ± 0.03	0.47 ± 0.03	0.63 ± 0.11	0.40 ± 0.03
TC (mmol/L)	2.49 ± 0.07	5.65 ± 0.23*	3.36 ± 0.34**	2.99 ± 0.14**
HDL-c (mmol/L)	1.05 ± 0.02	1.28 ± 0.03*	1.20 ± 0.02*	1.12 ± 0.01**
LDL-c (mmol/L)	2.07 ± 0.06	5.18 ± 0.23*	2.86 ± 0.31**	2.59 ± 0.14**
Insulin (ng/mL)	41.39± 9.28**	197.25 ± 24.46	64.91± 15.96**	76.62 ± 9.27**
HOMA-IR	0.51± 0.06**	3.71 ± 0.40	0.94± 0.12**	1.28 ± 0.12**

Mean ± SE, *p < 0.05 vs CO, **p < 0.05 vs HF

*TG* triglycerides, *TC* total cholesterol, *HDL-c* high density lipoprotein cholesterol, *LDL-c* low density lipoprotein cholesterol, *HOMA-IR* homeostatic model assessment insulin resistance

Figure 1 showed that obesity was confirmed after 8 weeks. Body weight was significantly lower in all groups compared to the HF group (*p* < 0.05). Also, DE group were significantly lower than the DC and CO groups (*p* < 0.05). Visceral fat was significantly lower in all groups compared to the HF group (*p* < 0.05). DE group was significantly lower than the DC group (*p* < 0.05).

**Fig. 3:**
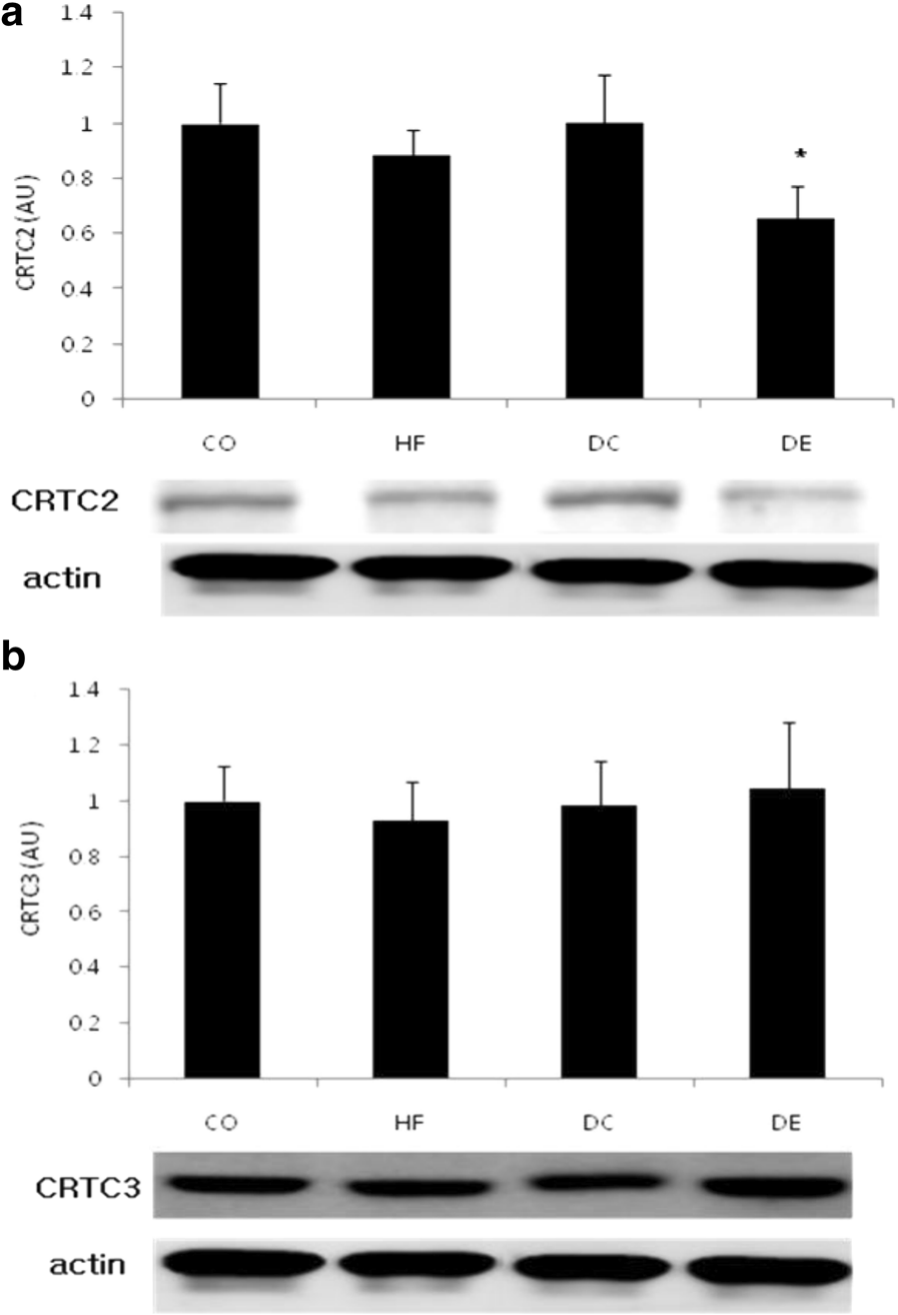
Change of CRTC family after exercise and diet. Mean ± SE, *p < 0.05 vs CO, #*p* < 0.05 vs HF

Concerning abdominal fat CRTC 2, the value in the DE group was significantly lower (35 %) than the CO group (*p* < 0.05), but did not differ from the HF group and DC group. For CRTC 3, there were no significant differences between the CO group, HF group, DC group, and DE group (Fig. 3).

**Fig. 4:**
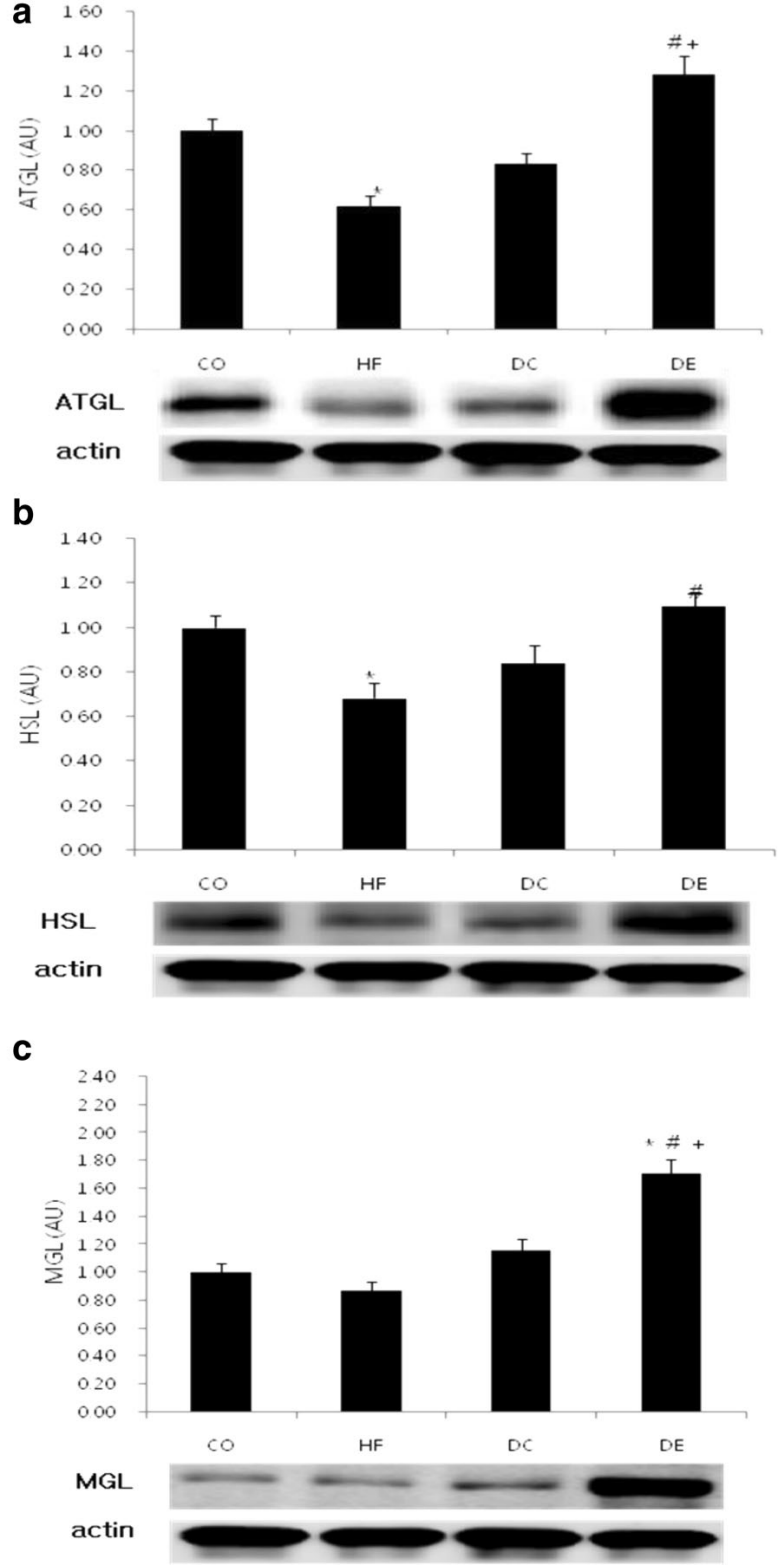
Change of lipolysis after exercise and diet. Mean ± SE, *p < 0.05 vs CO, #*p* < 0.05 vs HF, +*p* < 0.05 vs DC

Concerning abdominal fat ATGL, no appreciable difference was evident between the DC group and the CO group. A significantly lower (38 %) level was evident in the HF group (*p* < 0.05), whereas the DE group had a significantly higher (48 %) level than the HF group and the DC group (*p* < 0.05). In the case of HSL, there was no difference between the DC group and the DE group compared to the CO group, but HSL was significantly lower (32 %) in the HF group compared to the DE group only (*p* < 0.05). Finally, in the case of MGL, there were no differences between the CO group, HF group, and DC group. However MGL was significantly higher in the DE group (70 %) (*p* < 0.05) (Fig. 4).

## Discussion

The study investigated the effects of regular exercise and diet changes on the change in metabolic processes of the cAMP-Response Element-Binding Protein-Regulated Transcription Coactivator (CRTC) family and its sub-lipolysis. As the major finding of this study, there was no change in the expression of CRTC in the abdominal fat of mice continually fed a high fat diet even after being induced for obesity, but the protein expression of adipose tissue CRTC 2 was reduced in mice with a combined application of change to general diet and exercise. In addition, while the protein expressions of lipase ATGL and HSL were reduced in the mice fed with the high fat diet continually after obesity was induced, it was prominently increased in the general diet and exercise group. These results indicate that the combination of dietary change and exercise is more effective on lipolysis activity and energy consumption in the induced obesity model.

CRTC 1 regulates leptin expression in the hypothalamus [1], CRTC 2 triggers gluconeogenesis in the liver, which drives insulin resistance and preludes obesity [2, 3]. CRTC 3 inhibits use of fat burning in association with catecholamine in adipocytes [4]. Activation of CRTC genes suppresses lipolysis, which will impede energy consumption in turn driving obesity. Based on its role as a gluconeogenic regulator, CRTC 2 may be useful for treatment of hyperglycemia. Acute disruption of CRTC 2 is likely to reduce circulating glucose and triglyceride levels in insulin resistance [9–12]. Supportive evidence has been offered by a study conducted in CRTC 2 knockout mice, which verified that the reduced levels of triglyceride and improved insulin sensitivity [3]. Interestingly, adipose tissue CRTC 2 level was not changed by induction of obesity in the present study, but was significantly reduced by diet change and exercise treatment, whereas the expression of lipases (ATGL, HSL, MGL) in adipose tissue was significantly increased.

In the process of lipolysis, the stimulation of adenylyl cyclase due to the triggering action of a parent regulator, catecholamine, generates cAMP. Increased cAMP stimulates PKA, which activates ATGL [13]. ATGL catalysis produces hydrolysis of TAG and its conversion to DAG and MAG in a reaction catalyzed by HSL. MAG is broken down to fatty acids and glycerol by MGL [10]. The fatty acids and glycerol enter the circulatory system [6]. CRTC 2 has been functionally linked primarily to gluconeogenesis in the liver. Declined CRTC 2 expression in the liver activates lipolysis [14]. However, in this study the expression of lipase was increased as the expression of CRTC 2 was reduced in adipose tissues by diet change and exercise.

As CRTC 2 dephosphorylation sufficiently induces upregulation of glucogenic genes, such as phosphoenolpyruvate carboxykinase and glucose-6-phosphatase, and increases glucose production, the glucose level is normally maintained at a high level so that the lipase activity is likely to be inhibited [11–14]. However, the decreased glucose production noted when CRTC 2 expression was reduced might induce lipase activity as compensation for energy generation

Lipolysis begins by the stimulation of catecholamine signaling and resulting reduced lipid utilization, which promotes obesity. It is likely that CRTC 3 inhibits adenyl cyclase activity by reducing catecholamine signaling through regulator induction of the G protein signaling 2 gene [15–18]. The present study demonstrates that adipose tissue CRTC 3 was not changed by obesity or by diet change and exercise, while CRTC 2 was reduced by diet change and exercise and lipase expression was increased. Catecholamine signaling may be stimulated even without reduction of CRTC 3, which could contribute to improved lipolysis in white adipose tissue and improved fatty acid oxidation in brown adipose tissue.

Why diet change and exercise responded more sensitively to CRTC 2 than CRTC 3 concerning the expression of adipose tissue CRTC is still unclear. However, regular exercise reduces fat through energy consumption [7], diet change, and exercise in addressing high fat diet-induced obesity [19, 20]. Our previous study that treadmill training for 8 weeks and dietary changes did not have a positive impact on endocannabinoid system [21]. However, another study that regular exercise is effective in upregulating the mRNA expression of both myokine and angiogenesis factors in the skeletal muscle [22].

## Conclusion

To conclude, catecholamine secretion caused by exercise is independent of CRTC 3 activity. Stimulation of PKA may activate lipases, resulting in release of fatty acid from the adipocytes into the circulatory system, driving subsequent reduction of TAG. A simple diet that excludes exercise will not likely produce a change in adipose tissue CRTC family or activation of lipase.

## Abbreviations

ATGL: Adipose triglyceride lipase
CREB: cAMP response element binding protein
CRTC: Regulated transcription coactivator
DAG: Diacylglycerol
DC: Diet change group
DE: Diet change + exercise group
HDL-c: High density lipoprotein cholesterol
HF: High fat diet group
HOMA-IR: Homeostasis model assessment estimate of insulin resistance
HSL: Hormone sensitive lipolitic enzyme
LDL-c: Low density lipoprotein cholesterol
MAG: Monoacylglycerol
MGL: Monoacylglycerol lipase
PKA: Protein kinase A
TAG: Trigcylglycerol
TC: Total cholesterol
TG: Triglyceride

## References

[1] Altarejos JY, Goebel N, Conkright MD, Inoue H, Xie J, Arias CM, Sawchenko PE, Montminy M (2008). The Creb1 coactivator Crtc1 is required for energy balance and fertility. Nature Med.

[2] Saberi M, Bjelica D, Schenk S, Imamura T, Bandyopadhyay G, Li P, Vargeese C, Wang W, Bowman K, Zhang Y, Polisky B, Olefsky JM (2009). Novel liver-specific TORC2 siRNA corrects hyperglycemia in rodent models of type 2 diabetes. Am J Physiol Endocrinol Metab.

[3] Wang Y, Inoue H, Ravnskjaer K, Viste K, Miller N, Liu Y, Hedrick S, Vera L, Montminy M (2010). Targeted disruption of the CREB coactivator Crtc2 increases insulin sensitivity. Proc Natl Acad Sci U S A.

[4] Song Y, Altarejos J, Goodarzi MO, Inoue H, Guo X, Berdeaux R, Kim JH, Goode J, Igata M, Paz JC, Hogan MF, Singh PK, Goebel N, Vera L, Miller N, Cui J, Jones MR (2010). CHARGE Consortium; GIANT Consortium, Chen YD, Taylor KD, Hsueh WA, Rotter JI, Montminy M. CRTC3 links catecholamine signalling to energy balance. Nature.

[5] Lafontan M, Langin D (2009). Lipolysis and lipid mobilization in human adipose tissue. Prog Lipid Res.

[6] Altarejos JY, Montminy M (2011). CREB and the CRTC co-activators: sensors for hormonal and metabolic signals. Nat Rev Mol Cell Biol.

[7] Hawley JA, Lessard SJ (2008). Exercise training-induced improvements in insulin action. Acta Physiol (Oxf).

[8] Yeo NH, Shin KO, Jang KS, Bae JY, Woo SH, Kang S (2013). Effects of treadmill exercise on ECS(endocannabinoid) of central and peripheral tissue in high fat diet induced obesity mice. Korean J Sports Sci.

[9] Luo Q, Viste K, Urday-Zaa JC, Senthil Kumar G, Tsai WW, Talai A, Mayo KE, Montminy M, Radhakrishnan I (2012). Mechanism of CREB recognition and coactivation by the CREB-regulated transcriptional coactivator CRTC2. Proc Natl Acad Sci U S A.

[10] Hogan MF, Ravnskjaer K, Matsumura S, Huising MO, Hull RL, Kahn SE, Montminy M (2015). Hepatic insulin resistance following chronic activation of the CREB Coactivator CRTC2. J boil Chem.

[11] Koo SH, Flechner L, Qi L, Zhang X, Screaton RA, Jeffries S, Hedrick S, Xu W, Boussouar F, Brindle PK, Takemori H, Montminy MR (2005). The CREB coactivator TORC2 is a key regulator of fasting glucose metabolism. Nature.

[12] Dentin R, Hedrick S, Xie J, Yates J, Montminy MR (2008). Hepatic glucose sensing via the CREB Coactivator CRTC2. Science.

[13] Screaton RA, Conkright MD, Katoh Y, Best JL, Canettieri G, Jeffries S, Guzman E, Niessen S, Yates JR, Takemori H, Okamoto M, Montminy MR (2004). The CREB coactivator TORC2 functions as a calcium and cAMP sensitive coincidence detector. Cell.

[14] Le Lay J, Tuteja G, White P, Dhir R, Ahima RS, Kaestner KH (2009). CRTC2(TORC2) contributes to the transcriptional response to fasting in the liver but is not required for the maintenance of glucose homeostasis. Cell Metab.

[15] Hardy OT, Czech MP, Corvera S (2012). What causes the insulin resistance underlying obesity?. Curr Opin Endocrinol Diab Obes.

[16] Nagle CA, Klett EL, Coleman RA (2009). Hepatic triacylglycerol accumulation and insulin resistance. J Lipid Res.

[17] Bruno NE, Kelly KA, Hawkins R, Bramah-Lawani M, Amello AL, Nwachukwu JC, Nettles KW, Conkright MD (2014). Creb coactivators direct anabolic responses and enhance performance of skeletal muscle. EMBO J.

[18] Tripathy S, Torres-Gonzalez M, Jump DB (2010). Elevated hepatic fatty acid elongase-5 activity corrects dietary fat-induced hyperglycemia in obese C57BL/6 J mice. J Lipid Res.

[19] Kang S, Kim KB, Shin KO (2013). Exercise training improves leptin sensitivity in peripheral tissue of obese rats. BBRC.

[20] Woo J, Shin KO, Park SY, Jang KS, Kang S (2013). Effects of exercise and diet change on cognition function and synaptic plasticity in high fat diet induced obese rats. Lipids Health Dis.

[21] Yeo NH, Shin KO, Jang KS, Bae JY, Woo SH, Kang S (2013). Effects of treadmill exercise on ECS(endocannabinoid) of central and peripheral tissue in high fat diet induced obesity mice. Korean J Sport Sci.

[22] Shin KO, Bae JY, Woo J, Jang KS, Kim KS, Park JS, Kim IK, Kang S (2015). The effect of exercise on expression of myokine and angiogenesis mRNA in skeletal muscle of high fat diet induced obese rat. J Exer Nutr Biochem.

